# The comparison of sexual function in types I and II of female genital mutilation

**DOI:** 10.1186/s12905-023-02860-9

**Published:** 2024-01-08

**Authors:** Kosar Hassannezhad, Firouzeh Asadzadeh, Sohrab Iranpour, Soheila Rabiepoor, Pouran Akhavan Akbari

**Affiliations:** 1 Department of Midwifery, School of Nursing and Midwifery, Ardabil University of Medical Sciences, Ardabil, Iran; 2 Department of Community Medicine, School of Medicine, Ardabil University of Medical Sciences, Ardabil, Iran; 3 Professor of Reproductive health, Reproductive Health Research Center, Clinical Research Institute, Urmia University of Medical Sciences, Urmia, Iran; 4 Social Determinants of Health Research Center, Ardabil University of Medical Sciences, Ardabil, Iran

**Keywords:** Female genital mutilation, Circumcision, Female sexual function, Iran

## Abstract

**Background:**

Female genital mutilation has many sexual, physical, and psychological consequences. The present study aimed to examine the relationship between Female Genital Mutilation/Cutting (FGM/C), and Sexual Function among circumcised women in Sardasht City, Iran.”

**Methods:**

In this present cross-sectional study, 197 women who were mutilated entered the study by simple random sampling from two healthcare centers in Sardasht, Iran. A gynecologist first performed a genital examination to identify the type of female genital mutilation of participants. Subsequently, Socio-demographic and FGM/C-related characteristics checklist and the female sexual function index questionnaire were completed by interview method. Data were analyzed using SPSS 23 software.

**Results:**

Type I and II of female genital mutilation were performed in 73.1 and 26.9% of the participants, respectively. The age range of performing female genital mutilation in type I and II of female genital mutilation was 4–10 years old in 67.4% and 71.1% respectively. Traditional practitioners/local women carried out the circumcision in all of the participants, and Sunnah/tradition was reported as the most common reason for doing this procedure. The average total score of FSFI index in type I and II of female genital mutilation was 23.5 ± 2.0 and 17.4 ± 2.39, respectively. In all domains of FSFI, women with type II of female genital mutilation obtained lower scores than women with type I.

**Conclusion:**

Circumcised women have reduced scores in all domains of FSFI, and the severity of sexual dysfunction is related to the type of FGM/C. Considering the prevalence of female genital mutilation and its adverse effects, it is imperative to initiate cultural improvements through education and awareness. By educating and raising awareness among individuals about this issue, we can foster positive changes and address the problem effectively.

## Introduction

Female genital mutilation“(FGM/C) is one type of violence against women [1], which refers to all procedures related to the partial or total removal of the female external genitalia or damage to other genital organs for non-medical reasons. This painful procedure induced permanent physical, mental, and sexual problems in women who have undergone this practice [2]. World Health Organization classifies FGM/C into four groups: type I is partial or total removal of the clitoris, type II is partial or total removal of the glans clitoris and labia minor with or without removal of the labia major. Type III, also known as infibulation involves narrowing the vaginal opening by cutting and moving the small or large labia and sometimes sewing with or without the type of genital mutilation. Procedures such as pricking, piercing, incising, scraping, and cauterizing the genital region, as well as any others that cause injury to the female genitalia, fall under Type IV [1]. FGM/C is mainly carried out by non-physicians, such as traditional practitioners with religious or cultural motivations, which is associated with acute, mid-term, and long-term complications [3]. Pain, bleeding, urinary disorders, infection, pelvic inflammatory disease, infertility, and sexual dysfunction are among the most common consequences of FGM/C [1, 4].

Although female genital mutilation/circumcision is recognized as a violation of human rights at the international level, this practice was performed on at least 200 million girls and women in 31 countries on three continents of the world [5]. FGM/C is more prevalent in specific ethnic communities residing in Asia, Africa, and portions of the Middle East, such as Iran, Yemen, Oman, and the United Arab Emirates [6]. Rates of prevalence differ considerably between and within nations. Somalia had the highest FGM/C prevalence among women (99.2%), and Mali had the highest among girls (72.7%). The most common type of FGM/C among women was “flesh removed” (Type I or II) in 19 countries [7]. The prevalence of female circumcision in the United Arab Emirates was 41.4% in the study of Al Awar et al. and FGM/C prevalence in types I, II, and III was 62.8%, 16.6%, and 3.5%, respectively [8]. In Egypt, the prevalence of circumcision types I and II was reported at 73.6% and 26.4%, respectively, and Sexual dysfunction was identified in most women [9]. A recent meth-analysis study showed that among girls, “not sewn closed” (Type I, II, or IV) and “flesh removed” (Type I or II) were the most common types in 8 countries, respectively [7].

In Iran in the survey of Ahadi et al. in 2003, the prevalence of FGM/C was reported as 70% overall, and type I (87.4%) was more prevalent than type II (12.6%) [10]. In western cities of Iran, such as Ravansar and Kamyaran, the prevalence of FGM/C was reported at 55.7% [11] and 50.3% [12] respectively. Moreover, another study in south of Iran named Qeshm island was reported 60% [13].

FGM/C is associated with decreased sexual satisfaction [14]. Alinia et al. study in Kermanshah showed that the health utility value of FGM/C in women with type I is significantly lower than in women with type II circumcision. Furthermore, FGM/C affects the sexual and psychological health of women, reduces personal and marital satisfaction, and ultimately leads to a decrease in quality of life-related to their health [15].

The cultural and traditional components of FGM/C are various in different ethnic groups [16]. Reducing recklessness, and ensuring virginity before marriage are cultural and religious reasons for justifying female circumcision [17]. FGM/C threatens sexual and mental health [18]. Women who have undergone FGM/C have sexual disorders, such as dyspareunia, lower vaginal lubrication, sexual pain, lack of sexual desire, and experience less orgasm [14, 19]. Both types of FGM/C can be associated with sexual dysfunction [9]. The research done in Piranshahr revealed that there is a higher prevalence of sexual dysfunction among women who have had circumcision. However, no statistically significant differences were seen in terms of the overall quality of life between circumcised and uncircumcised women [20]. Considering traditional context and cultural considerations of Kurdish areas about FGM/C and limited studies regarding the comparison of different types of FGM/C in Iran, the present study was conducted to compare the sexual function in types I and II of Female Genital Mutilation /Cutting (FGM/C) among circumcised women in Sardasht City, Iran.

## Materials and methods

This cross-sectional descriptive study was conducted in Sardasht, from June to September 2022. Sardasht city is located in the western Kurdish region of Iran.197 reproductive-age women with a history of FGM/C referred to two public Gynecological clinics in Sardasht were studied. Participants were selected by the convenience sampling method.

The inclusion criteria in this study were married, of reproductive age, not pregnant and not lactating, history of performing genital mutilation, continuous sexual activity at least once a month, no history of Psychological, metabolic disease, and hormonal disorders affecting sexual activities, no history of sexual/physical assaults, not known sexual dysfunction in the spouse and no Surgery of female genital organs.

The sample size was determined 205 women, using Power G software with a power of 80%, an error level of 5% (a = 0.5), and an effect size of 0.08 and 9 predictor variables. Regarding the lack of an accurate statistical population, sampling was calculated as available. To determine the type of FGM/C, first, eligible women who consented to participate in the study were examined by a gynecologist, and the study’s objectives were explained to them. Upon securing written permission from the subjects, the researcher proceeded to administer the Checklists of socio-demographic and FGM/C-related information, as well as the FSFI questionnaire. The FSFI questionnaire was developed by Rosen in 2000 [21]. Female Sexual Function Index (FSFI) examines female sexual function in the last four weeks. It has 19 questions in six domains: sexual desire, sexual arousal, lubrication, orgasm, satisfaction, and pain. The sub-scales in question include a spectrum of responses ranging from 0 to 1 to 5. Higher scores on these sub-scales are indicative of enhanced sexual function. The highest score attainable for each sub-scale is 6, while the overall scale has a maximum score of 36. A score of zero indicates no sexual activity during the last four weeks The cut-off points of FSFI subscales in arousal, lubrication, and orgasm domains were 3.4, satisfaction and pain 3.8, and desire domain was reported 3.3. In 2011, Ahmad Fakhri et al. studied the validity and reliability of this questionnaire, reporting the reliability coefficient by Cronbach’s alpha method (0.86), and intra-cluster correlation coefficient (0.77), and the appropriate Cut-off point of the scale for screening sexual dysfunction was determined to be 28 [22]. The reliability of the Persian version of questionnaire was assessed by Cronbach’s alpha coefficient and was obtained at 0.82. The collected data were entered into SPSS 23 software and analyzed as descriptive and inferential statistics.

## Results

Eight of the 205 women who had undergone genital mutilation were subsequently excluded from the study for non-compliance and failure to complete the questionnaires; thus, the final sample size consisted of 197 women. The sociodemographic characteristics of the participants are detailed in Table 1. In terms of the characteristics of FGM/C, 73.1% and 26.9% of the participants were diagnosed as type I and II FGM/C, respectively. The average age of the participating women in type I and II of FGM/C was 30.9 ± 8.7 and 30 ± 8.6 years old, respectively, and the average duration of marriage was 12.5 ± 0.8 and 12.66 ± 0.6 years in type I and II of FGM/C, respectively. All participants and their spouses were Sunni in religion. The age range of most participants in practicing time of FGM/C in type I and II (67.4% and 71.1% respectively) was 4–10 years old. Traditional practitioners/local women carried out the circumcision in all of the participants and Sunnah/tradition was reported as the most common reason for doing this procedure (67.4% and 73.6% in type I and II respectively). The majority of participants were illiterate, housewives, lived in rural areas, which had insufficient economic income. Natural family planning methods including rhythmic and withdrawal methods were used by women with type 1 and II FGM/C as contraception (Table 1).

**Table 1:** Socio-demographic characteristics of participants

Variable	Type of variable	Type of FGM		*P* value
		Type I No. (percent)	Type II No. (percent)	
FGM/C type	Type I/ II	144(73.1)	53(26.9)	-
Education	Illiterate	73 (50.7)	32 (60.3)	0.598
	Sub diploma	58 (40.2)	16 (30.2)	
	Diploma	9 (6.3)	3 (5.7)	
	Bachelor and above	4 (2.8)	2 (3.8)	
Spouse`s Education	Illiterate	82 (56.9)	26 (49.1)	0.685
	Sub diploma	29 (20.1)	14 (26.4)	
	Diploma	20 (13.9)	9 (17)	
	Bachelor and above	13 (9.0)	4 (7.5)	
Residency	City	52 (36.1)	23 (43.4)	0.350
	Rural areas	92 (63.9)	30 (56.6)	
Residency status	Rental	87 (60.4)	31 (58.5)	0.968
	Paternal	29 (20.1)	11 (20.8)	
	Personal	28 (19.4)	11 (20.8)	
Income level	Insufficient	55 (38.2)	26 (49.1)	0.271
	Fair enough	44 (30.6)	16 (30.2)	
	Sufficient	45 (31.3)	11 (20.8)	
Occupation	housewife	125 (86.8)	46 (86.8)	0.415
	Employee	8 (5.6)	5 (9.4)	
	Self-employed	11 (7.6)	2 (3.8)	
Spouse`s Occupation	Employee	18 (12.5)	9 (17.0)	0.417
	Self-employed	126 (87.5)	44 (83.0)	
Type of delivery	vaginal	99 (68.8)	33 (62.3)	0.391
	C section	45 (31.3)	20 (37.7)	
Contraception method	Pill	42 (29.2)	16 (30.2)	0.682
	Condom	14 (9.7)	9 (17)	
	Contraceptive injection	4 (2.8)	1 (1.9)	
	Natural family planning methods	72 (50)	25 (47.2)	
	tubal ligation	7 (4.9)	1 (1.9)	
	Vasectomy	2 (1.4)	1 (1.9)	
	No methods	3 (2.1)	0 (0.0)	
Number of delivery	Nulliparous	6 (4.2)	2 (3.8)	0.676
	2≥	88 (61.1)	36 (67.9)	
	3≤	50 (34.7)	15 (28.3)	
FGM/C age range(year)	0–4	32(22.2)	8(15.1)	0.560
	4–10	97(67.4)	38(71.7)	
	10–15	8(5.6)	5(9.4)	
	15–20	7(4.9)	2(3.8)	
The reason for FGM/C performing	Tradition	97(67.4)	39(73.6)	0.598
	Chastity	31(21.5)	8(15.1)	
	Both	16(11.1)	6(11.3)	
	Other	0	0	
Age	Mean ± St.Deviation	30.88 ± 8.79	30.03 ± 8.63	0.546
Duration of marriage	Mean ± St.Deviation	12.49 ± 0.8.3	12.66 ± 8.59	0.901

chi-square and independent sample t-tests were used for data analysis

The average score of FSFI in type I and II of FGM/C was 23.5 ± 2.0 and 17.4 ± 2.39, respectively, and in all domains of FSFI, women with type II of female genital mutilation obtained lower scores than women with type I. Data analysis showed a significant difference in FSFI total score and all subscales between two groups of women who were mutilated (*p* = 000). Based on the cut-off point of each sub-scale, an investigation of the FSFI in type I and type II FGM/C revealed disorders linked to the sub-domains of desire (34. % and 75.5 respectively), arousal (29.9% and 71.1% respectively), lubrication (13.2% and54.7 respectively), orgasm (20.1%and 67.9% respectively), satisfaction (43.8 and 79.2% respectively) and pain (39.6% and 94.3% respectively) (Table 2).

**Table 2:** Comparison of total score of FSFI and it’s domains in type I and II FGM/C

Variables	FGM/C type I		FGM/C type II		*P* value
	Disorder	Mean ± St.Deviation	Disorder	Mean ± St.Deviation	-
	Number (percent)		Number (percent)		
Desire	49 (34)	3.5 ± 0.95	40 (75.5)	2.4 ± 1.0	0.000
Arousal	43 (29.9)	3.7 ± 0.71	38 (71.1)	3.0 ± 1.0	0.000
Lubrication	19 (13.2)	4.0 ± 0.74	29 (54.7)	3.4 ± 0.97	0.000
Orgasm	29 (20.1)	4.2 ± 0.84	36 (67.9)	3.0 ± 0.86	0.000
Satisfaction	63 (43.8)	3.9 ± 0.72	42 (79.2)	2.6 ± 1.1	0.000
Pain	57 (39.6)	3.9 ± 0.92	50 (94.3)	2.7 ± 1.4	0.000
FSFI	142(98.6)	23.5 ± 2.0	53(100)	17.4 ± 2.4	0.000

An Independent t-test sample was used for data analysis

## Discussion

The present study was conducted with the aim of “the comparison of female sexual function in types I and II of Female Genital Mutilation “, and 197 circumcised women were studied. Similar to Ismail study, Type I and II of female genital mutilation had been performed in 73.1 and 26.9% of the participants, respectively. The age range of most participants in practicing time of FGM/C in type I and II (67.4% and 71.1% respectively) was 4–10 years old.

The average score of FSFI in 99% of circumcised women was below 28, and they were screened for sexual dysfunction. The average score of FSFI in type I and II of FGM/C was 23.5 ± 2.0 and 17.4 ± 2.39, respectively, and in all domains of FSFI, women with type II of FGM/C obtained lower scores than women with type I. The average score of FSFI in type I and II of FGM/C indicates more severe sexual dysfunction in women with type II.

Sardasht is one of the Kurdish cities is located in the west of Iran where most people have Sunni religion, and FGM/C has been commonly performed among women living there from past decades.

It is necessary to explain that Islam is classified into several branches. Sunni Islam is by far the largest branch of Islam. The name Sunni Islam is derived from the term Ahl al-Sunna wa-l-Jama`a (principles of Sunnah and Prophetic community). Sunni Islam claims to guide Muslims on the path of the Prophet’s teachings and habits [23].

The results of the present study showed that more than half of the participating women and their husbands in both groups of FGM/C were illiterate, lived in a village, and had Low income. The majority of women were housewives. Regarding the education and occupation status of women who had been circumcised, in the study conducted in Egypt, more than 80% of women with FGM/C were illiterate and unemployed [24]. Additionally, our results are consistent with a research by Pashaei et al. showed that daughters of low-literate moms who live in rural regions and have a favorable attitude about FGM/C are more likely to conduct it. They let it happen to their daughters because they are under societal pressure [25]. Low literacy levels among the rural people were stated as a reason for girls’ mutilation in the Daneshkhah et al. study in Iran [20]. FGM/C is accepted as a social norm in this area, and most illiterate people, mainly illiterate mothers, support this practice. Along with education, low income can play a role as a predictor factor to FGM/C performance too. In Guinea women with secondary/higher education, whose partners had secondary/higher education and rich women were less likely to circumcise their daughters [26].

The effect of mothers’ and societies’ positive attitudes and beliefs towards FGM/C and the social acceptance of this practice was proved in some studies [25, 27], so that Gambian girls who did not perform mutilation were addressed with the derogatory titles of sinners, insolent, impure, and shameless [27]. The majority of teenagers and their parents in traditional societies lack access to accurate information due to cultural reasons [28], so increasing women’s educational attainment and raising public awareness of FGM/C and its effects, particularly in rural areas, may help lower the rate of female circumcision. Based on WHO report, FGM/C is mainly carried out on young girls between infancy and age 15 and occasionally on adult women [1]. The age range of FGM/C performance in the majority of participants in both types of FGM/C was 4–10 years and there were no significant differences between the two groups of FGM/C (I and II). Some studies reported various age ranges for FGM/C performing performance, for example before the age of 7 in the study conducted in Ravansar [25], and before the age of 3 years in the Biglo et al. study [29]. Based on the studies, it is concluded that in communities with a culture of circumcision, this practice is usually performed from infancy to adolescence, that is, before marriage. Most of the girls in these communities get married in a teenage period.

Consistent with Dehghan Khalili and his colleague’s research, the reason for all types of FGM/C in our study was traditional cultural beliefs [30]. The reasons for FGM vary from place to region and throughout time, and include a combination of sociocultural variables within families and communities [1]. Religious motivation was the most significant factor for FGM/C in Biglo’s research [29]. However, in Oljira et al.‘s study, mothers of the girls mentioned social acceptance and the possibility of a better marriage as the main reason to perform FGM/C and it was significantly related to the mother’s age, education level, and FGM/C history in women’s of family [28].

Sardasht people recognize FGM/C as a tradition named Sunna and believe that this practice causes chastity in women. Since tradition is one of the causes of FGM/C, living in rural areas where tradition is more respected, increases the prevalence of FGM/C. Circumcision seems to have more ancient roots than religious ones, and it is not included in the Muslim holy book, the Qur’an. Religious leaders can be crucial in raising awareness of this issue and changing people’s beliefs to lessen female circumcision in Sunni societies because the practice of FGM predates both Islam and Judaism and is widespread among both religious and non-religious groups [31], and performing FGM/C is not religious instruction, so religious leaders can play an important role in emphasizing this issue and modifying people’s beliefs in order to reduce female circumcision in Sunni societies.

Similarly, to Biglo [29] and Oljira’s research [28], the current study’s findings indicate that local/traditional circumcisers performed both type I and II of FGM/C. Female circumcision is not recognized as a medical procedure, so this practice is often performed by local women in non-sterile conditions, and mostly without anesthesia. Depending on the type of circumcision, it has different complications and risks for women.

Inconsistent with the Abdi Karim and Alsibiani et al. study, in which the frequency of type II FGM/C was more than type I [31, 32], our study’s results indicated that most participating women had undergone type I FGM/C and there was no case of type III and IV circumcision. However, Ismail et al. [9] and Piroozi et al. had similar results to our study [18]. Although all forms of FGM are associated with an increased risk of health complications, the risk is greater with more severe forms of FGM.

The total FSFI score average of participants and separately for women with type I and II FGM/C were obtained at 21.87 ± 3.44, 23.5 ± 2.0 and 17.4 ± 2.4, respectively, which is in line with other studies [9, 32, 33], Although the total FSFI score in Manal’s survey, 14.3 ± 5.93, was less than our study. Compared to uncircumcised women, a significant decrease in FSFI score and sexual performance has been proven in women who experienced FGM/C [29, 34].

Pain (39.6 and 94.3% in type I and II respectively) and satisfaction disorders (43.8 and 79.2% in type I and II respectively) were the most common screened disorders in our study, also circumcised women were usually less capable of sexual arousal [35]. In all domains of FSFI, women with type II FGM/C obtained lower scores than women with type I (Table 2), and there was a significant difference between two groups. Disorders in all domains of FSFI was identified in Daneshkhah et al. [20], and Manal Ibrahim Mahmoud’s studies [33], but the comparison of two types of FGM/C was not done in their study. In Somali women, only scores of the orgasm and satisfaction domains were lower in Type II compared to type I [32]. Contrary to our findings, the sexual pain scores were not affected in other studies [31, 36], it could be due to the different versions of used questionnaires (Arabic versus Persian) and comparing circumcised women with uncircumcised ones not two types of FGM/C.

Sexual dysfunction was more common in type II FGM/C women, since there was a substantial correlation between the two groups [32, 34]. Type III circumcision has the poorest sexual consequences, and women with type I to III FGM/C have sexual dysfunction [37]. The type of circumcision was associated with Medical complications, such as bleeding, sexual disorders, and delivery complications [8]. More severe sexual dysfunction in women with type II circumcision is related to the nature of the surgery and irreparable damage to the female reproductive system and requires rehabilitation interventions to reduce sexual problems in these women. Sexual desire disorder is a sustainable complication in women who were mutilated [38] and can lead to dissatisfaction with sexual life [39]. A decrease or lack of sexual desire can induce an inability to gender role-playing [40] and worrying about losing the spouse in women [41], which requires more attention from sexual health service providers.

## Conclusion

Sexual dysfunction is common among women with FGM/C, and its severity is associated with the type of FGM/C. Low awareness of illiterate women and men, especially those who live in rural areas about FGM/C and its consequences, increases their acceptance of circumcision. In order to mitigate the negative consequences associated with FGM/C, it is imperative to implement cultural transformations and behavioral adjustments via education and awareness campaigns. Thus, necessary interventions should be considered to reduce sexual and psychological complications in circumcised women with all types of FGM/C.

## Data Availability

The datasets generated and analyzed during the current study are available from the corresponding author.

## Declarations

### Author(s) Statement

Consent was obtained from all subjects and their legal guardian(s).
